# Prevalence of human papillomavirus among Wenzhou women diagnosed with cervical intraepithelial neoplasia and cervical cancer

**DOI:** 10.1186/s13027-018-0211-8

**Published:** 2018-11-26

**Authors:** Chanqiong Zhang, Chongan Huang, Xiang Zheng, Dan Pan

**Affiliations:** 1 Department of Pathology, Wenzhou People’s Hospital, The third Clinical Institute Affiliated to Wenzhou Medical University, 299 Guan Rd. Ouhai district, Wenzhou, Zhejiang, China; 2 Department of Spine Surgery, The Second Affiliated Hospital and Yuying Children’s Hospital of Wenzhou Medical University, Wenzhou, Zhejiang, China

**Keywords:** Human papilloma virus, Cervical intraepithelial neoplasia, Genotype distribution

## Abstract

**Background:**

Human papillomavirus (HPV) is associated with an increased risk of cervical cancer. Using a vaccine to prevent HPV infections could be a cost-effective strategy to decrease the incidence of cervical cancer. Learning about the characteristics of CIN patients with HPV infection in Wenzhou is a key step in guiding the use of HPV vaccines and screening for cervical cancer.

**Methods:**

We undertook a retrospective analysis including 2612 women who were treated in the Department of Gynecology and Obstetrics from Jan 2016 to Nov 2017. All of the patients were examined by HPV testing and histology.

**Results:**

The prevalence of HR-HPV among women with cervical intraepithelial lesions aged 65–69 years (38.8%) was significantly higher than that of the other age groups. The percentage of patients diagnosed with HPV-positive HSIL progressively increased with age to a maximum of 18.0% in the group of 40 to 44 years of age. HPV 16, 52, and 58 were the three most dominant genotypes among these women, and single infections (950, 73.3%) were more common than multiple infections (346, 26.7%). Compared to cervicitis, the odds ratios (ORs) for LSIL associated with HPV 33, 52, 16 and HPV 58 infection were 5.98, 3.91, 3.65, 3.65, and 3.188, respectively; for HSIL associated with HPV 16, 33, 58 and HPV 31 were 9.30, 7.68, 5.97, and 4.21, respectively. In LSIL, the frequencies of HR-HPV 52,16,58,18 were 19.3,18.2,10.9, and 7.8%, respectively.

**Conclusion:**

Our study provides important data about the HPV genotype distribution and its correlation with cervical intraepithelial lesions in the Wenzhou population.

## Introduction

Cervical cancer is the third most common type of cancer among women and has the fourth highest mortality rate worldwide [1]. Recent estimates showed that 102,000 new cases of cervical cancer were diagnosed in China in 2014, with a crude incidence rate of 15.30/100000 [2].

Human papillomavirus (HPV) infection has shown to play a key role in the pathogenesis of cervical cancer. However, most HPV infection are transient and can resolve spontaneously. Persistent cervical HPV infections are the main cause of cervical precancerous lesions and cervical cancer in a long-lasting multistage process [3]. Premalignant and malignant endocervical glandular lesions are much more uncommon than their squamous counterparts in the cervix, but are increasing in prevalence [4, 5]. Though the incidence of cervical adenocarcinoma (ADC) has increased over the past several decades in young women, SCC is the predominant histological type among all cervical cancers and the principal component of the trend [6]. Early detection and management of cervical intraepithelial neoplasia is of great significance in reducing the incidence of cervical cancer.

Among more than 200 HPV genotypes discovered, nearly 20 genotypes belong to the high-risk HPV (HR-HPV) and probable high-risk categories [7]. There are, however, significant viral characteristics and regional differences in the HPV genotype distribution.

It has previously been observed that cervical intraepithelial neoplasia (CIN) is a premalignant cervical disease caused by high-risk human HPV (HR-HPV) infection. HPV 16 and 18 account for 65–75% of cervical cancers, while 12 other high-risk genotypes account for the remaining [8–10]. The development of preventive and therapeutic vaccines has become an important measure to decrease the incidence and mortality of cervical cancer [11, 12].

Extensive research has shown that vaccines against HR-HPV are highly safe, well tolerated, with very few severe side effects when given to adolescent and preadolescent females prior to first sexual intercourse [13]. Since 2006 the first HPV vaccine in the world, the 4-valent HPV (4vHPV) vaccine against HPV 6, 11, 16, and 18, has been licensed by the Food and Drug Administration (FDA) [14]. In June 2017, the CFDA approved Gardasil®‘s entry into the Chinese market. After the value of the four-valent vaccine was confirmed, researchers set out to develop new vaccines to prevent the five other common oncogenic HPVs, including HPV 31, 33, 45, 52, and 58. Since 2014, a 9-valent HPV (9vHPV) vaccine has been available to provide protection against HPV 6, 11, 16, 18, 31, 33, 45, 52, and 58 [15]. Recently, the nine-valent vaccine was approved by the China Food and Drug Administration (CFDA).

The detection of the HPV genotypes has a pivotal role in the diagnosis, clinical treatment, and prognosis of cervical cancer. Wenzhou is the largest city in Zhejiang Province, with a population of more than 9 million. However, the distribution of HR-HPV genotypes in female residents with cervical precancerous lesions and cancer in Wenzhou has rarely been evaluated. In this study, HR-HPV infection rates and HPV genotype distribution were evaluated in 2612 patients living in Wenzhou who were diagnosed with cervicitis, CIN1, CIN2/3, or Cervical squamous cell carcinoma (SCC). This study aimed to contribute to this growing area of research by exploring characteristics of HPV infection and the correlation between cervical intraepithelial neoplasia and HPV infection. These results will help to improve risk stratification of HR-HPV-positive women in cervical screening programs.

It is important to determine the epidemiology of mono- and coinfections of HPV in order to establish appropriate prevention strategies for the design of novel vaccines based on each population. Determining the epidemiology of mono- and coinfections of HPV has been instrumental in establishing appropriate prevention strategies for the design of new vaccines tailored to each population [16–18].

## Materials and methods

### Population

Retrospectively, this study included 2612 outpatients and inpatients visiting the Department of Gynecology at Wenzhou People’s Hospital between Jan 2016 and Nov 2017 for cervical diseases. Primary exclusion criteria for the study were: patients with adenocarcinoma; patients currently pregnant; patients with a history of total uterine or cervical resection; patients with prior chemotherapy or radiation treatment for cervical neoplasia; patients with previous physical treatment of the cervix or hormone treatment for cervical disease; or patients with known human immunodeficiency virus infection. The women taking part in the study ranged in age from 18 to 96 years, and the mean age was 44 years. Among these participants, 1855 were diagnosed with cervicitis, 390 with CIN1, 297 with CIN2/3, and 70 with cervical SCC. The age, clinical gynecological, cytological, and histopathologic data as well as HPV testing results of the study participants were recorded. This study was approved by the Hospital Ethics Committee of the Wenzhou People’s Hospital. As a retrospective study, written patient consent was not required.

### HPV testing

HPV genotyping was carried out using a flow cytometry fluorescence hybridization method. Samples were prepared according to the manufacturer’s instructions. HPV testing was performed using PCR technology on a Luminex 200 system with an HPV nucleic acid detection kit (Tellgen Corporation, Shanghai, China) to identify 17 high-risk types (16, 18, 31, 33, 35, 39, 45, 51, 52, 56, 58, 59, 66, 68, 26, 53, and 82), and 10 low-risk types (6, 11, 40, 42, 43, 44, 55, 61, 81, and 83) [19, 20]. The HPV DNA chip used in this study was capable of detecting multiple HPV subtypes simultaneously and showed higher sensitivity and specificity than the sensitivity and specificity of traditional cytology, fluorescence in situ hybridization and PCR. Positive and negative control samples were included in each experiment.

### Histological analysis

All participants were subjected to gynecological examinations and cervical scraping for cytological examination. Those patients with epithelial cell abnormalities of atypical squamous cells of undetermined significance (ASC-US) or higher cytological grade were referred for colposcopy examination and biopsy. Histopathology was diagnosed by two senior pathologists. In the analysis, we included CIN1 in the LSIL group and CIN2/3 in the HSIL group according to the latest classification (4th WHO Women’s Reproductive System Tumor Classification). All patients with CIN 1 having LSIL, along with all patients with CIN grade 1+. Additionally CIN 2+ and CIN 3 were categorized as HSIL.

### Statistics

The distribution of HPV genotypes was analyzed by the stratification of Bethesda system-based cervical histology, and patient age was divided into 5-year groups. For comparing the groups, we utilized the χ^2^ test or Fisher’s exact test. *P* < 0.05 was considered statistically significant. The odds ratio (ORs) and relative 95% confidence intervals (CIs) were analyzed with IBM SPSS version 21.0. The significance level α was set at 0.05.

## Results

A total of 2612 patients were recruited for this study with an average age of 44 years. Overall, cervicitis was confirmed in 71% (1855/2612), LSIL was diagnosed in 14.9% (390/2612), HSIL in 11.4% (297/2612) and cervical carcinomas in 2.7% (70/2612) by histological analysis.

Table 1 shows the HPV prevalence among 2612 women stratified by 10-year age groups. The women in the ≤29 years group had the highest positive rate for HPV (80.7%) followed by patients aged 30–34 (70.1%), 35–39 (59.1%), 65–69 (55.1%), 40–44 (49.6%), 60–64 (45.0%), 55–59 (41.2%), ≥70 (37.7%), 50–54 (35.6%), and 45–49 (32.0%).

**Table 1:** Distribution of age and HPV prevalence in enrolled patients

characteristics	HPV positive rate in 2612 women	HR-HPV rate in CIN group
Age		
≤ 29	209(80.7%)	92(35.5%)
30–34	211(70.1%)	106(35.2%)
35–39	182(59.1%)	103(33.4%)
40–44	221(49.6%)	124(27.8%)
45–49	166(32.0%)	89(17.1%)
50–54	149(35.6%)	76(18.2%)
55–59	54(41.2%)	27(20.6%)
60–64	54(45.0%)	32(26.7%)
65–69	27(55.1%)	19(38.8%)
≥ 70	23(37.7%)	19(31.1%)
HPV		
Single-type infection	950(73.3%)	
Multiple-type infection	346(26.70%)	
2 types	252(72.80%)	
3 types	68(19.70%)	
4 types	19(5.50%)	
5 types	5(1.40%)	
6 types	2(0.60%)	
HR-multiple	218(63.00%)	
LR-multiple	8(2.30%)	
HR-LR multiple	120(34.70%)	

CIN, cervical intraepithelial neoplasia. HR-multiple, multiple high-risk human papillomavirus. LR-multiple, multiple low-risk human papillomavirus. HR-LR multiple, multiple high-risk combined with low risk human papillomavirus

The prevalence rates of HR-HPV among women with cervical intraepithelial lesions were different for each age group, with one peak at 65–69 years (38.8%), which was significantly higher than the rates of the other age groups (30–34, 35–39, 40–44, 50–54, or 60 years; Table 1) (*P* < 0.0001). The percentage of HPV-positive HSIL patients progressively increased with age to a maximum of 18.0% in patients aged 40 to 44 years. There was a significant difference between that age group (40–44 years) and all other age groups (30–34, 35–39, 40–44, 50–54, or 60 years; Table 1).

Among these women, the majority (49.6%) of patients were infected with HR- and/or LR- HPV. Single infections (950, 73.3%) were more common than multiple infections (346, 26.7%) in both the any-HPV genotype group and the HR-HPV group. Table 1 also shows the comparative distribution of multi-infections. The most common situation was a two-type infection (72.8%), followed by a three-types infection (19.7%), four-types infection (5.5%), five-types infection (1.4%), and six-types infection (0.6%). Furthermore, multitype infections were divided into HR-multiple infections, LR-multiple infections and HR-LR multiple infections. The percentage of HR-multiple infections accounted for more than 63% of all multi-HPV infections in the study. In contrast, LR-multiple infections (2.3%) and HR-LR multiple infections (34.7%) were found in multi-HPV cases. The prevalence of LR-multiple infections was substantially lower than that for HR-multiple infections (*P* < 0.001).

**Fig. 1:**
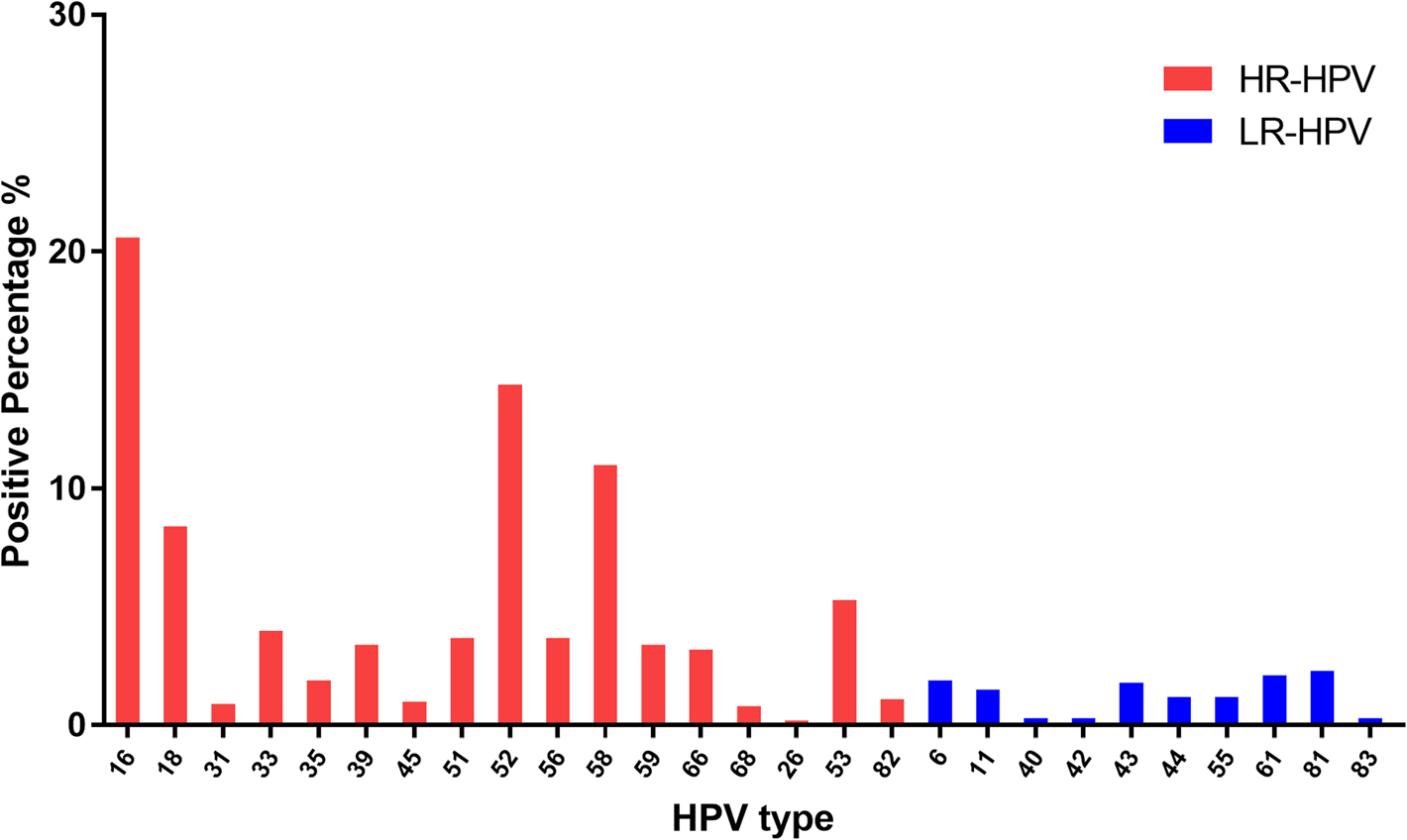
Distribution of HR- and LR-HPV types among HPV-positive women. HR-HPV includes HPV types 16, 18, 31, 33, 35, 39, 45, 51, 52, 56, 58, 59, 66, 68, 26, 53, and 82. LR-HPV includes HPV types 6, 11, 40, 42, 43, 44, 55, 61, 81, and 83. HPV, human papillomavirus; HR, high-risk; LR, low-risk

Among the women participating in the study, 1296 were screened and shown to have an HPV infection. As clearly seen in Fig. 1, among the detected types of HPV, HR-HPV 16 was the most frequent type (20.6%), followed by HR-HPV 52 (14.4%), HR-HPV 58 (11.0%), HR-HPV 18 (8.4%), HR-HPV 53 (5.1%), and HR-HPV 33 (4.3%).

**Fig. 2:**
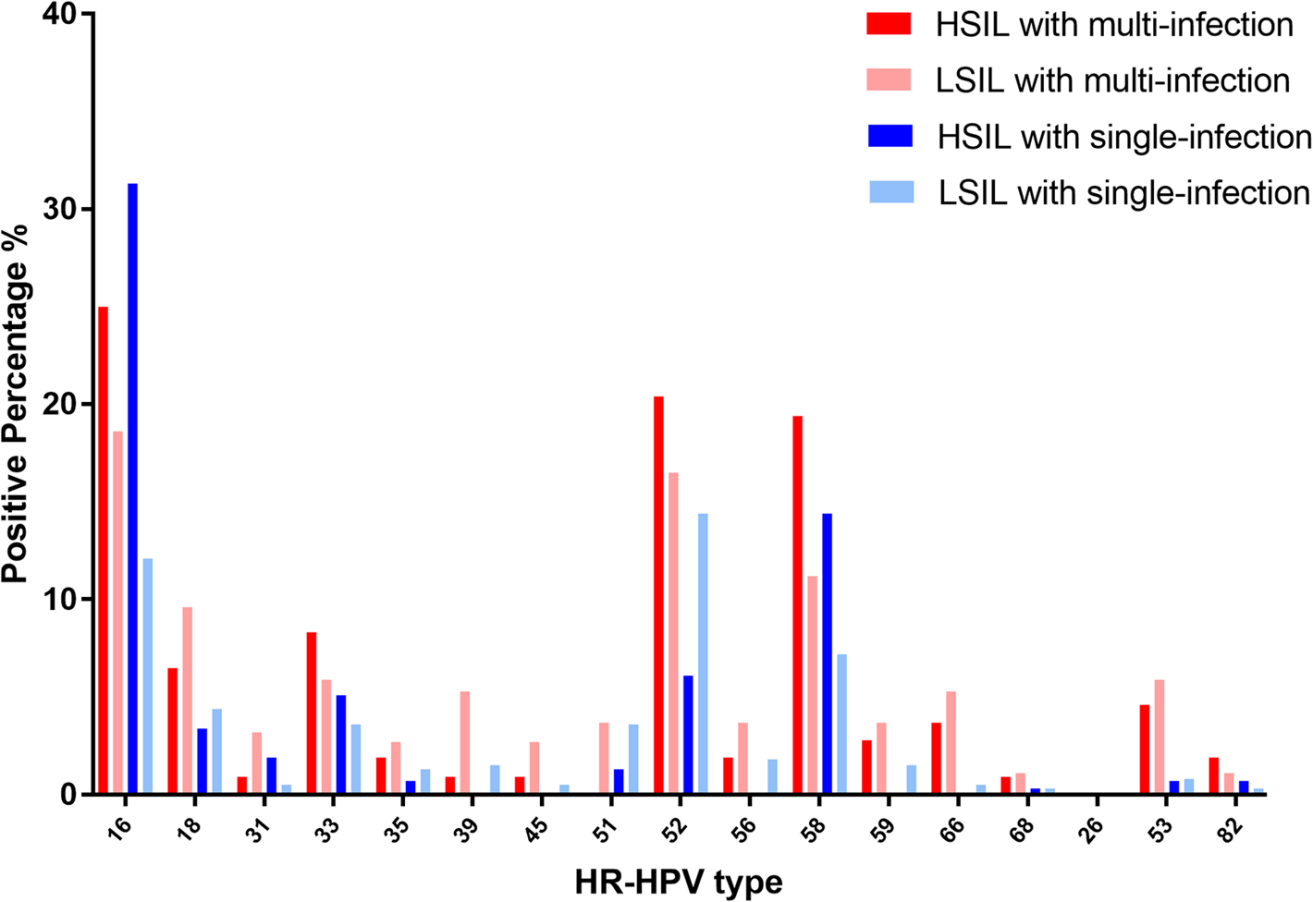
Relative distribution of HR-HPV genotypes among LSIL and HSIL groups. LSIL: low-grade squamous intraepithelial lesions, include CIN 1; HSIL: high-grade squamous intraepithelial lesions, include CIN2/3

The number of LSIL and HSIL cases with a single HPV infection is shown in Fig. 2. HPV 16 (31.3%), 58 (14.1%), 52 (6.1%), and 33 (5.1%) were the most frequent types found in HSIL. In comparison, HPV 52, 16, 58, and 18 caused the majority of LSIL cases with attribution rates of 14.4, 12.1, 7.2, and 4.4%, respectively. The prevalence and attribution of the top four genotypes varied greatly between LSIL and HSIL.

As shown in Fig. 2, multiple infections were analyzed in LSIL and HSIL. Similarly, HPV 16 (25.0%), 52 (20.4%), 58 (19.4%), and 33 (8.3%) also ranked in the top four in HSIL. In LSIL, HPV 16, 52, 58, and 18 were the most common types with attribution rates of 18.6, 16.5, 11.2, and 9.6%, respectively.

The HPV-positive rates of patients with cervicitis, LSIL, HSIL, and cervical SCC are displayed in Table 2. The frequency of HPV infections among the 297 HSIL cases (84.8%) was higher than that in any other group. The prevalence of single-type infection in HSIL was 65.3%, in cervical SCC was 62.9%, in LSIL was (56.2%), and in cervicitis (26.6%). The percentage of positive multi-infections accounted for 24.4% in LSILs, 19.5% in HSIL, 10.1% in cervicitis and 8.6% in cervical SCC.

**Table 2:** Prevalence of HPV genotypes by cervical histology

	n	HPV Positive	Single-infection	Multi-infection
Cervictis	1855(73.0%)	680(36.7%)	493(26.6%)	187(10.1%)
LSIL	390(15.3%)	314(80.5%)	219(56.2%)	95(24.4%)
HSIL	297(11.7%)	252(84.8%)	194(65.3%)	58(19.5%)
Cervical cancer	70(2.7%)	50(71.4%)	44(62.9%)	6(8.6%)

LSIL: low-grade squamous intraepithelial lesion,include CIN 1; HSIL: high-grade squamous intraepithelial lesion,include CIN2/3

To determine the association between HR-HPV and cervical intraepithelial neoplasia, we calculated the ORs and 95% CIs by using a logistic regression models. As presented in Table 3, LSIL had a strong relationship with HPV 33 (OR = 5.98, 95% CI = 3.31–10.80), HPV 52 (OR = 3.91, 95% CI = 2.90–5.27) and HPV 16 (OR = 3.65, 95% CI = 2.70–4.95), HPV 58 (OR = 3.188, 95% CI = 2.19–4.63). HSIL was significantly associated with single-type infection with HPV 16 (OR = 9.30, 95% CI = 6.93–12.48), 33 (OR = 7.68, 95% CI = 4.22–14.0), and 58 (OR = 5.97, 95% CI = 4.18–8.54), 31 (OR = 4.21, 95% CI = 1.18–15.0). The corresponding values for cervical SCC were significantly associated with HPV 16 (OR = 15.39, 95% CI = 9.30–25.44).

**Table 3:** Relationship between different HPV types in LSIL,HSIL,Cervical cancer

HPV type	n	OR	95%CI		*P* value
			Lower limt Upper limit		
LSIL					
HPV52	87	3.907	2.898	5.267	<0.001
HPV16	82	3.653	2.697	4.948	<0.001
HPV58	49	3.188	2.194	4.634	<0.001
HPV18	35	1.911	1.273	2.869	*P* = 0.003
HPV33	25	5.982	3.313	10.801	<0.001
HPV51	21	2.582	1.505	4.431	*P* = 0.001
HPV31	6	4.815	1.545	15.009	*P* = 0.01
HPV35	10	2.686	1.23	5.864	*P* = 0.02
HPV39	16	1.761	0.983	3.154	*P* = 0.059
HPV45	7	4.825	1.683	13.835	*P* = 0.005
HPV56	14	1.344	0.736	2.456	*P* = 0.318
HPV59	13	1.489	0.791	2.8	*P* = 0.209
HPV66	12	1.44	0.749	2.772	*P* = 0.268
HPV68	3	1.43	0.392	5.221	*P* = 0.482
HPV53	14	0.922	0.515	1.652	*P* = 0.855
HPV82	3	1.098	0.312	3.873	*P* = 0.749
HSIL					
HPV52	40	2.118	1.45	3.093	*P*<0.001
HPV16	120	9.303	6.934	12.483	*P*<0.001
HPV58	63	5.974	4.179	8.538	*P*<0.001
HPV18	17	1.177	0.691	2.006	*P* = 0.566
HPV33	24	7.678	4.217	13.98	*P*<0.001
HPV51	4	0.619	0.22	1.744	*P* = 0.507
HPV31	4	4.207	1.18	14.998	*P* = 0.038
HPV35	4	1.393	0.468	4.146	*P* = 0.532
HPV39	1	0.139	0.019	1.013	*P* = 0.016
HPV45	1	0.886	0.109	7.226	*P* = 1
HPV56	2	0.245	0.059	1.011	*P* = 0.039
HPV59	3	0.44	0.136	1.43	*P* = 0.194
HPV66	4	0.619	0.22	1.744	P = 0.507
HPV68	2	1.251	0.273	5.737	*P* = 0.676
HPV53	7	0.598	0.272	1.312	*P* = 0.244
HPV82	4	1.934	0.626	5.973	*P* = 0.278
Cervical cancer					
HPV52	1	0.197	0.027	1.432	*P* = 0.085
HPV16	37	15.386	9.304	25.441	*P*<0.001
HPV58	3	0.993	0.306	3.227	*P*>0.05
HPV18	5	1.491	0.586	3.793	*P*>0.05

LSIL: low-grade squamous intraepithelial lesion,include CIN 1; HSIL: high-grade squamous intraepithelial lesion,include CIN2/3. OR, odds ratio

The proportion of HPV genotypes detected according to cervical histology are given in Table 4. HR-HPV 52 was the most frequently detected genotype in normal histology cases (13.6%). HR-HPV 16 (13.5%) and HPV 18 (9.7%) were also the most common types detected in normal histology cases. In patients with LSIL, HR-HPV 52 was the most frequently detected type (19.3%), followed by HR-HPV 16 (18.2%), HR-HPV 58 (10.9%), and HR-HPV 18 (7.8%). HR-HPV 16 was the most frequently detected type in HSIL (36.7%), and the next most frequent types were HR-HPV 58 (19.3%) and HR-HPV 52 (12.2%). The percentage of patients with HPV 16 detected among the HR-HPV genotypes was 66.1%, and it was markedly higher in the cervical SCC group than in the other groups.

**Table 4:** HPV genotypes in cases with histological of diagnosis of cervicitis, LSIL, HSIL and cervical cancer

HPV type	Cervicitis	LSIL	HSIL	cervical cancer	*P* value
16	126(13.5%)	82(18.2%)	120(36.7%)	37(66.1%)	*P*<0.001
18	91(9.7%)	35(7.8%)	17(5.2%)	5(8.9%)	*P*>0.05
31	6(0.6%)	6(1.3%)	4(1.2%)	0	*P*>0.05
33	21(2.2%)	25(5.6%)	24(7.3%)	0	*P*<0.001
35	18(1.9%)	10(2.2%)	4(1.2%)	1(1.8%)	*P*>0.05
39	44(4.7%)	16(3.6%)	1(0.3%)	0	*P* = 0.001
45	7(0.7%)	7(1.6%)	1(0.3%)	2(3.6%)	*P*>0.05
51	40(4.3%)	21(4.7%)	4(1.2%)	0	*P* = 0.019
52	127(13.6%)	87(19.3%)	40(12.2%)	1(1.8%)	*P*<0.001
56	50(5.4%)	14(3.1%)	2(0.6%)	0	*P*<0.001
58	80(8.6%)	49(10.9%)	63(19.3%)	3(5.4%)	*P*<0.001
59	42(4.5%)	13(2.9%)	3(0.9%)	3(5.4%)	*P* = 0.015
66	40(4.3%)	12(2.7%)	4(1.2%)	0	*P* = 0.018
68	10(1.1%)	3(0.7%)	2(0.6%)	0	*P*>0.05
26	3(0.3%)	0(0.0%)	0(0.0%)	0	*P*>0.05
53	72(7.7%)	14(3.1%)	7(2.1%)	1(1.8%)	*P*<0.001
82	13(1.4%)	3(0.7%)	4(1.2%)	0	*P*>0.05
6	21(2.2%)	6(1.3%)	3(0.9%)	0	*P*>0.05
11	17(1.8%)	8(1.8%)	1(0.3%)	1(1.8%)	*P*>0.05
40	4(0.4%)	1(0.2%)	1(0.3%)	0	*P*>0.05
42	5(0.5%)	0(0.0%)	0(0.0%)	0	*P*>0.05
43	16(1.7%)	12(2.7%)	3(0.9%)	0	*P*>0.05
44	12(1.3%)	4(0.9%)	5(1.5%)	0	*P*>0.05
55	17(1.8%)	3(0.7%)	2(0.6%)	0	*P*>0.05
61	20(2.1%)	8(1.8%)	10(3.1%)	0	*P*>0.05
81	28(3.0%)	10(2.2%)	2(0.6%)	1(1.8%)	*P*>0.05
83	4(0.4%)	1(0.2%)	0(0.0%)	1(1.8%)	*P*>0.05

LSIL: low-grade squamous intraepithelial lesion,include CIN 1; HSIL: high-grade squamous intraepithelial lesion,include CIN2/3

## Discussion

Cervical cancer is one of the most common malignant tumors affecting women in our country, the development process may involve a long-term, persistent HPV infection that induces gradual changes of cervical squamous epithelium from mild to highly abnormal hyperplasia, eventually leading to malignant transformation, in approximately 10 years or longer [21]. Epidemiological studies suggest that 70% of cervical cancer cases are related to HPV 16 and HPV 18 infections [22]. Available vaccines protect against two, four, or nine types of HPV. All vaccines protect against at least HPV type 16 and 18 that have the greatest risk for cervical cancer.

Screening for HPV-infected patients is of profound significance for clinical treatment and prevention of cervical malignant transformation. Therefore, learning about the characteristics of HR-HPV prevalence in Wenzhou could not only contribute to guiding HPV vaccine development and use but also guide effective measures to prevent and control the incidence of cervical intraepithelial lesions and cervical cancer.

The current study provided additional information on the relative distribution of HR-HPV genotypes among cervical lesions in Wenzhou, China. We found that HPV 16, 52, 58, 18, and 53 are the most common types among women with persistent HR-HPV infections in Wenzhou. A large number of studies have proven that HR-HPV has a high prevalence rate in cervical cancer and cervical intraepithelial lesions. Wang et al. previously reported that in a retrospective analysis the 5 most common HR-HPV genotypes were HPV 16, 58, 52, 33 and 18, in descending order of prevalence, which is similar to our findings [20]. The distribution of HPV types shows substantial differences among LSIL and HSIL patients. HPV 16, 58, and 52 were the top three common types in HSILs, but the rank of the top three types was different in LSILs. These results are consistent with the findings of a great deal of previous works [23]. In concordance with previous research, our data reported that the HR-HPV prevalence was correlated with the severity of CIN.

When we classified cervical histology by age, among the HR-HPV-positive population, those 65–69 years old had the highest rate. In Gu Y’s report, 20.6, 14.9, 15.9, 17.4, and 21.2% of subjects in the age ≤ 24, 25–34, 35–44, 45–55, and > 55 groups were high-risk HPVs positive, respectively [24]. However, this outcome is contrary to that of Wang et al., who found the prevalence peak of HR-HPV among cervical precancerous lesions occurred in the group aged 45 to 49 years [25].

To further investigate, we divided these women into cervicitis, LSIL, HSIL, and cervical SCC groups according to their pathological status. As a result, the HPV-positive rates of women with LSIL, HSIL and cervical SCC were significantly higher than the rate in patients with cervicitis. In accordance with the present results, previous studies have demonstrated that the HPV infection rate increases as the level of cervical pathology status increases [26, 27].

When cases were divided into single and multiple-type infections, the difference in the positive rate between the two groups was significant, indicating that single infection accounted for the majority of HPV infections. The single-type infection rate increased as the cervical pathology status developed. Multiple HPV infections have reportedly shown a higher proportion of LSILs. These results are in agreement with those obtained by Chan et al. [26].

We found that HPV 33, 52 and 16 increased the risk for LSIL compared with cervicitis patients. Our data also supported the hypothesis that the prevalence of persistent HPV infection was also increased with the severity of the cervical lesion and showed HPV 16, 33, 58, and 31 were most frequent in HSIL cases. These results are consistent with those of Anderson LA, who found that the HR-HPV prevalence increased with the severity of CIN [28]. In Wang S′ research, there was a population-based study that supported the hypothesis that the prevalence of persistent HPV infection was also increased along with the severity of the cervical lesion and showed HPV 16, 58, 18, 52, and 33 were the most widespread in persistent infection.

The proportions of HPV genotypes detected according to cervical histology is given in Table 4. HR-HPV 52 was the most frequently detected genotype in cervicitis and LSIL cases. In patients with HSIL, HR-HPV 16 was the most common type in the HSIL and cervical SCC groups. Numerous studies in China have reported findings consistent with our results [29, 30]. Compared to the distribution of HR-HPV among HSILs worldwide, HPV 16 is always the most common type, which is consistent with our findings [31, 32].

The major limitation of the study is the fact that we could not access information on whether participants were vaccinated. In addition, the population distribution in our study was uneven, and we need to expand the sample size.

## Conclusion

Our study provides important data of HPV genotype distribution and correlation with cervical intraepithelial lesion in Wenzhou population. We shall confirm these results with prospective study and lager sample size.
